# The Senolytic Drug Navitoclax (ABT-263) Causes Trabecular Bone Loss and Impaired Osteoprogenitor Function in Aged Mice

**DOI:** 10.3389/fcell.2020.00354

**Published:** 2020-05-20

**Authors:** Anuj K. Sharma, Rachel L. Roberts, Reginald D. Benson, Jessica L. Pierce, Kanglun Yu, Mark W. Hamrick, Meghan E. McGee-Lawrence

**Affiliations:** ^1^Department of Cellular Biology and Anatomy, Medical College of Georgia, Augusta University, Augusta, GA, United States; ^2^Department of Orthopaedic Surgery, Augusta University, Augusta, GA, United States

**Keywords:** osteoblast, bone marrow stromal cell, skeleton, senescence, senolytic, osteoporosis

## Abstract

Senescence is a cellular defense mechanism that helps cells prevent acquired damage, but chronic senescence, as in aging, can contribute to the development of age-related tissue dysfunction and disease. Previous studies clearly show that removal of senescent cells can help prevent tissue dysfunction and extend healthspan during aging. Senescence increases with age in the skeletal system, and selective depletion of senescent cells or inhibition of their senescence-associated secretory phenotype (SASP) has been reported to maintain or improve bone mass in aged mice. This suggests that promoting the selective removal of senescent cells, via the use of senolytic agents, can be beneficial in the treatment of aging-related bone loss and osteoporosis. Navitoclax (also known as ABT-263) is a chemotherapeutic drug reported to effectively clear senescent hematopoietic stem cells, muscle stem cells, and mesenchymal stromal cells in previous studies, but its *in vivo* effects on bone mass had not yet been reported. Therefore, the purpose of this study was to assess the effects of short-term navitoclax treatment on bone mass and osteoprogenitor function in old mice. Aged (24 month old) male and female mice were treated with navitoclax (50 mg/kg body mass daily) for 2 weeks. Surprisingly, despite decreasing senescent cell burden, navitoclax treatment decreased trabecular bone volume fraction in aged female and male mice (−60.1% females, −45.6% males), and BMSC-derived osteoblasts from the navitoclax treated mice were impaired in their ability to produce a mineralized matrix (−88% females, −83% males). Moreover, *in vitro* administration of navitoclax decreased BMSC colony formation and calcified matrix production by aged BMSC-derived osteoblasts, similar to effects seen with the primary BMSC from the animals treated *in vivo*. Navitoclax also significantly increased metrics of cytotoxicity in both male and female osteogenic cultures (+1.0 to +11.3 fold). Taken together, these results suggest a potentially harmful effect of navitoclax on skeletal-lineage cells that should be explored further to definitively assess navitoclax’s potential (or risk) as a therapeutic agent for combatting age-related musculoskeletal dysfunction and bone loss.

## Introduction

Senescence is a cellular defense mechanism that helps cells prevent acquired damage; acute senescence can be beneficial in processes related to wound healing, injury repair, and development, but chronic senescence, as in aging, can contribute to the development of age-related tissue dysfunction and disease (Van Deursen, 2014; Calcinotto et al., 2019). Characteristic aspects of senescence include proliferative arrest, changes in chromatin organization, and an altered secretome referred to as the senescence-associated secretory phenotype, or SASP (Coppe et al., 2008).

It has been clearly documented that removal of senescent cells can help prevent tissue dysfunction and extend healthspan during aging. For example, inducible elimination of *p16^Ink4a^*-positive senescent cells delayed onset of age related pathologies and improved physical function in old (26–28 month) mice (Baker et al., 2011; Xu et al., 2018). Likewise, transplantation of senescent cells promoted physical dysfunction in young mice, whereas treatment of aged (20+ months old) mice with the senolytic drug combination of dasatinib + quercetin reduced senescent cell burden and improved physical function in aged mice (Xu et al., 2018). With specific regards to skeletal biology, expression of the *p16^Ink4a^* senescence marker increases with age in the skeletal niche (including bone marrow, osteoblast progenitors, osteoblasts, and osteocytes) (Farr et al., 2016). In particular, Osterix-expressing (Osx1+) osteoprogenitor cells decrease with age in mouse bone marrow, and the remaining Osx1+ cells in old mice show increased senescence (Kim et al., 2017). Critically, selective depletion of senescent cells via INK-ATTAC caspase 8 activation or inhibition of the SASP via JAK inhibitors reduced bone resorption activity and maintained trabecular bone mass in aged (20–22 month old) treated as compared to control mice (Farr et al., 2017). This suggests that promoting the selective removal of senescent cells, via the use of senolytic agents, can be beneficial in the treatment of aging-related bone loss.

Several compounds, including the dasatinib + quercetin senolytic drug combination mentioned above (Xu et al., 2018), have been tested to see the target effect on senescent cells and amelioration of aging or disease phenotypes (Grezella et al., 2018), with some even entering clinical trials (Hickson et al., 2019; Justice et al., 2019). However, many of these therapeutics have shown a high degree of cell and tissue specificity. Navitoclax (also known as ABT-263) is a chemotherapeutic drug whose role in senescent cells that was first described in 2016 (Chang et al., 2016; Zhu et al., 2016). It has been reported to target Bcl-2 family members including Bcl−2, Bcl−xl, and Bcl−w, promoting the apoptosis of senescent cells [which depend upon anti−apoptotic defenses similarly to cancer cells (Zhu et al., 2015)]. At least two reports suggest that unlike some of the more cell-type specific senolytic agents, navitoclax has a broad spectrum of activity across multiple human cell lines, although some disagreement exists (Chang et al., 2016; Zhu et al., 2016; Grezella et al., 2018). Regardless, navitoclax was previously reported to effectively clear senescent hematopoietic stem cells in the bone marrow and senescent muscle stem cells in the hindlimb of aged mice (Chang et al., 2016). In addition, navitoclax showed promise in depleting senescent cells from cultures of human and murine mesenchymal stromal cells (Kim et al., 2017; Grezella et al., 2018). However, navitoclax is also a chemotherapeutic agent, with reported toxic side effects including transient thrombocytopenia and neutropenia (Rudin et al., 2012; Kaefer et al., 2014), meaning its relative potential benefit *in vivo* as compared to risk factors was not yet known. Therefore, the purpose of this study was to assess the effects of short-term navitoclax treatment on bone mass and osteoprogenitor function in aged mice.

## Materials and Methods

### Animals and *in vivo* Administration of Navitoclax

All experiments followed NIH guidelines and were approved by the Institutional Animal Care and Use Committee at Augusta University. Male and female C57BL/6 mice (24 months of age, *n* = 10 females and *n* = 10 males) from the National Institute on Aging aged rodent colony were obtained for study. Mice were permitted water and standard rodent chow (RD: Teklad #2918) *ad libitum*, and were treated with the senolytic drug navitoclax (ABT-263, 50 mg/kg body mass, *n* = 5 per sex) or vehicle (10% ethanol, 30% PEG 400, 60% Phosal50, *n* = 5 per sex) once daily for 2 weeks by oral gavage. This dosage of navitoclax was previously shown to effectively clear senescent hematopoietic stem cells in the bone marrow and senescent muscle stem cells in the hindlimb of aged mice (Chang et al., 2016). At the conclusion of study, mice were sacrificed by carbon dioxide inhalation followed by thoracotomy. One femur per mouse was aseptically harvested for BMSC isolation as described previously (McGee-Lawrence et al., 2013). One tibia per mouse was fixed in 10% neutral buffered formalin, decalcified in 15% EDTA, paraffin embedded, histologically sectioned, and stained with hematoxylin and eosin for gross tissue visualization; one sample (female navitoclax treatment) was damaged during processing and was excluded from analysis. Trabecular bone volume fraction in the proximal tibia was quantified histologically (Dempster et al., 2013) using image analysis software (Bioquant Osteo, Nashville, TN, United States) to assess bone mass. Serial sections were treated with proteinase K and subjected to *in situ* TUNEL staining to detect apoptotic cells as previously described (McGee-Lawrence et al., 2013). Tissue sections were stained with a TUNEL detection kit (Roche #11 767 305 001, #11 767 291 910) according to the manufacturer’s protocol, mounted with DAPI-containing medium (Vectashield), and imaged via confocal microscopy to assess apoptotic cells. The percentage of apoptotic (TUNEL+ nucleus) cells in the bone marrow, normalized to tissue area, was quantified at 200X total magnification for each mouse using image analysis software (Bioquant Osteo).

### Isolation of Bone Marrow Stromal Cells (BMSC) From Treated Mice

Adherent bone marrow stromal cells (BMSC) from the navitoclax or vehicle-treated mice were isolated as previously described (McGee-Lawrence et al., 2013). Briefly, bone marrow was flushed from the diaphysis, and BMSC were immediately seeded into 6-well plates (10 million cells per well) in osteogenic medium (alpha MEM + 20% FBS + 1% antibiotic/antimycotic + 50 μg/ml ascorbic acid + 10 mM beta glycerophosphate + 100 nM dexamethasone). Cells were cultured for 21 days prior to assessment of mineralized matrix production by alizarin red staining (*n* = 3 wells per group) as previously described (McGee-Lawrence et al., 2013). The fractional area of each well covered with alizarin red-stained calcified matrix was quantified with image analysis software (Bioquant Osteo, Nashville, TN, United States). Parallel cultures were harvested after 7 days of growth for semi-quantitative PCR-based analysis of gene expression (*n* = 3 wells per group; males only due to limitations in the number of cells obtained for female mice). Total RNA was extracted and purified from the cultures with TRIzol reagent (Invitrogen) and reverse transcribed into cDNA using the SuperScript III First-Strand Synthesis System (Invitrogen). Relative transcript levels of the early osteoblastic genes Runx2 (NM_009820, Forward 5-GGCACAGACAGAAGCTTGATGA-3′, Reverse 5-GAATGCGCCCTAAATCACTGA-3′) and Osterix/Sp7 (NM_130458, Forward 5′-GGAGGTTTCACTCCATTCCA-3′, Reverse 5′-TAGAAGGAGCAGGGGACAGA-3′) were quantified and normalized to expression of Gapdh (NM_008084, Forward 5′-GGGAAGCCCATCACCATCTT-3′, Reverse 5′-GCCTCACCCCATTTGATGTT-3′) as previously described (McGee-Lawrence et al., 2013). Reactions were performed using 37.5 ng of cDNA per 15 μl with QuantaBio Perfecta SYBR Green SuperMix (VWR) and the Bio-Rad CFX Connect Real Time PCR Detection System.

### Isolation of Bone Marrow Stromal Cells (BMSC) for *in vitro* Navitoclax Treatment

To observe the direct effects of navitoclax on BMSC, primary BMSC were isolated from aged wildtype mice and treated with navitoclax (or vehicle) *in vitro*. An additional cohort of 24 month old male and female wildtype C57BL/6J mice was obtained from the NIA aged rodent colony (*n* = 5 per sex). In addition, for follow-up studies on TUNEL staining, a group of old female wildtype mice (27 months of age, *n* = 5) was culled from an in-house breeding colony [wildtype Osx1-Cre-negative Hdac3^fl/fl^ mice (McGee-Lawrence et al., 2016, 2018)]. For both groups of mice, BMSC were isolated as described above and previously (McGee-Lawrence et al., 2013). Mice were sacrificed by carbon dioxide inhalation followed by thoracotomy, and long bones (femurs, tibias, humerii) were aseptically harvested. Bone marrow was flushed from the long bone diaphyses, and adherent BMSC were cultured as described in each assay below.

### Colony Formation and Mineralized Matrix Production

Bone marrow stromal cells were seeded into 12 well plates (4 million cells per well) in growth medium (alpha MEM + 20% FBS + 1% antibiotic/antimycotic), or osteogenic cell culture medium (alpha MEM + 20% FBS + 1% antibiotic/antimycotic + 50 ug/mL ascorbic acid + 10 mM beta glycerophosphate) with or without dexamethasone. Dexamethasone is a common osteogenic additive which is required for osteogenic differentiation of human BMSCs (Beresford et al., 1994) and that is routinely included in osteoblast cultures from other species as well [although not strictly required for murine stromal cells (Lecka-Czernik et al., 1999)]. Dexamethasone was included in cultures to best replicate experiments conducted with BMSC isolated from the mice treated *in vivo*, but as glucocorticoids like dexamethasone can affect matrix mineralization, apoptosis, and mechanisms of lipid storage by osteoblasts (McGee-Lawrence et al., 2016; Nie et al., 2018; Deng et al., 2019), additional cultures were seeded in osteogenic medium with no dexamethasone to determine whether glucocorticoid inclusion affected the impact of navitoclax on the cells. Cells were grown for 21 days to promote colony formation as measured by crystal violet staining, or to promote formation of a mineralized extracellular matrix detectable by alizarin red staining (McGee-Lawrence et al., 2016). Navitoclax (5 μM) or vehicle (DMSO) was included in the culture medium for the entirety of the experiment. At the conclusion of studies, cells were fixed in 10% formalin and stained with crystal violet or 2% alizarin red, as described (McGee-Lawrence et al., 2013, 2016). The areal fraction of each well covered in crystal violet or alizarin red-stained calcified matrix was quantified with image analysis software (Bioquant Osteo; *n* = 3 wells per condition).

### Senescence-Associated Beta Galactosidase (SA-β gal) Staining

Bone marrow stromal cells were seeded in 100 mm dishes (70 million cells per dish) in growth medium (alpha MEM + 20% FBS + 1% antibiotic/antimycotic) or osteogenic culture medium (growth medium + 50 ug/mL ascorbic acid + 10 mM beta glycerophosphate) with or without 100 nM dexamethasone and cultured for 10 days, after which cells were trypsinized, resuspended, and seeded into 12-well plates (100,000 cells per well). After overnight attachment, culture medium was changed to include 5 μM navitoclax or vehicle (DMSO) for 5 days. Cells were fixed in 10% formalin and stained to detect senescence-associated beta galactosidase activity with a commercial kit (Cell Signaling Technology Senescence β-Galactosidase Staining Kit #9860) as per the manufacturer’s protocol to identify senescent cells (blue staining). Cells were imaged with a digital camera (Jenoptik) and inverted phase contrast microscope (Olympus IX-70), and the percentage of senescent cells normalized to total cell number for each condition was quantified from six random images per well and *n* = 6 wells per condition using image analysis software (Bioquant Osteo).

### Cell Viability – MTT Assay

Bone marrow stromal cells were grown as described above for SA-β gal experiments and seeded into 96 well plates (20,000 cells per well). After overnight attachment, culture medium was changed to include 5 μM navitoclax or vehicle (DMSO) for 5 days; a “no treatment” control (with no vehicle or navitoclax) was also included for calculation of relative cytotoxicity. Cellular metabolic activity was then assessed via measurement of conversion of water soluble MTT to insoluble formazan using a commercial MTT assay (abcam MTT Assay kit, #ab211091). Absorbance was measured at 590 nm in a multi-functional plate reader (Bio-Tek Cytation 5); 7 wells per group were averaged for each condition.

### TUNEL Staining

Bone marrow stromal cells were grown as described above for SA-β gal experiments and seeded into 12-well plates onto sterile glass coverslips (100,000 cells per well). After overnight attachment, culture medium was changed to include 5 μM navitoclax or vehicle (DMSO) for either 24 h or 5 days. DNA damage was assessed via *in situ* TUNEL staining as previously described (McGee-Lawrence et al., 2013). Briefly, cells were fixed with 4% paraformaldehyde, lysed with PBS containing 0.1% TritonX-100 and 0.1% sodium citrate, and stained with an *in situ* TUNEL detection kit (Roche #11 767 305 001, #11 767 291 910). TUNEL stained coverslips were mounted with DAPI-containing medium (Vectashield) and examined via confocal microscopy to assess apoptotic cells. The percentage of apoptotic (TUNEL+ nucleus) cells, normalized to total cell number, for each condition was quantified from *n* = 3 random images per coverslip and *n* = 2 coverslips per condition using image analysis software (Bioquant Osteo).

### Statistics

Statistical analyses were performed with JMP Pro 14.0.0 software (SAS Institute Inc., Cary, NC, United States). For qPCR data, fold changes were log transformed prior to analysis. Data were compared between groups within each experiment with Student’s *t*-tests (when only two groups were compared) or 2-factor ANOVA with interaction (factor 1: sex, factor 2: drug treatment) with Tukey-Kramer HSD *post hoc* multiple comparisons tests when significant interactions were detected. A significance of *p* < 0.05 was used for all comparisons.

## Results

### Navitoclax Administration Caused Trabecular Bone Loss *in vivo* and Impaired BMSC Function

**FIGURE 1:**
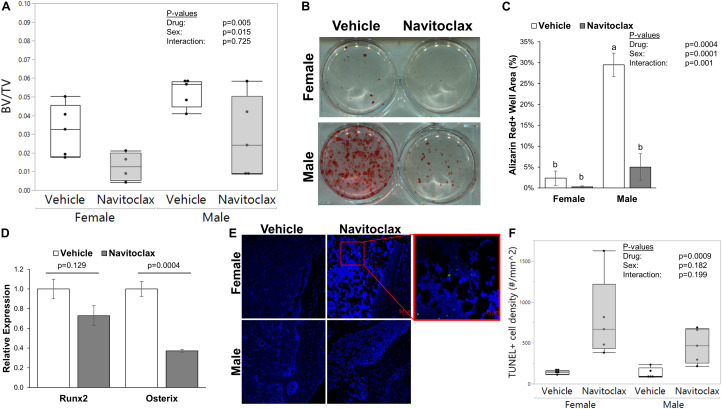
Navitoclax administration caused trabecular bone loss and impaired osteoprogenitor differentiation. **(A)** Navitoclax (50 mg/kg body mass, once daily) was administered to 24 month-old male and female mice via oral gavage for 2 weeks. Trabecular bone volume fraction, measured histologically in the proximal tibia, was significantly decreased by navitoclax treatment. **(B,C)** Primary BMSC from the mice **(A)** were harvested at sacrifice and subjected to osteogenic culture conditions for 21 days. Cells from navitoclax-treated mice demonstrated reduced production of calcified matrix **(C)**. **(D)** BMSC were cultured under osteogenic conditions for 7 days; cells from navitoclax-treated mice demonstrated reduced expression of the osteoprogenitor gene Osterix/Sp7. **(E,F)** Frontal sections of the tibia from each mouse were stained with an *in situ* TUNEL detection kit to quantify apoptosis in the bone marrow; TUNEL+ cells were identified by green staining and normalized to tissue area. Original magnification: 200X; inset box shows the region magnified to 630X. Box plots show the median, quartiles, and outlier fences for each dataset, where outlier fences represent first quartile –1.5*(interquartile range) and third quartile +1.5*(interquartile range); each data point shown represents one mouse. *P*-values shown represent statistical comparisons between groups, *p*-value for “drug” in 2-factor ANOVAs represents the comparison between navitoclax (gray) and vehicle (white)-treated animals. A Tukey-Kramer HSD *post hoc* multiple comparisons test was performed when significant interactions were detected; bars with different superscript letters are significantly (*p* < 0.05) different from one another.

It has been previously reported that selective removal of senescent cells was sufficient to maintain trabecular bone mass in aged mice (Farr et al., 2017). Surprisingly, 2 weeks of *in vivo* administration of navitoclax did not improve, and instead significantly decreased trabecular bone volume fraction in aged female and male mice (−60.1% females, −45.6% males) (Figure 1A). BMSC-derived osteoblasts from the navitoclax treated mice were impaired in their ability to produce a mineralized matrix (−88% females, −83% males), although pairwise comparison differences in females did not achieve statistical significance, likely because mineralized matrix was substantially lower overall in the BMSC cultures from females as compared to males (Figures 1B,C). BMSC-derived osteoblasts from the male navitoclax-treated mice demonstrated significantly lower expression levels of the osteoprogenitor gene Osterix/Sp7 (−63%), consistent with an impaired osteogenic phenotype (Figure 1D). TUNEL staining revealed increased TUNEL+ cells in the bone marrow of male and female navitoclax-treated mice (Figures 1E,F), consistent with navitoclax’s previously reported pro-apoptotic effects (Zhu et al., 2016).

### *In vitro* Treatment With Navitoclax Reduced Senescent Cell Burden, but Also Impaired Mineralized Matrix Production, Increased Cytotoxicity, and Increased Apoptosis

**FIGURE 2:**
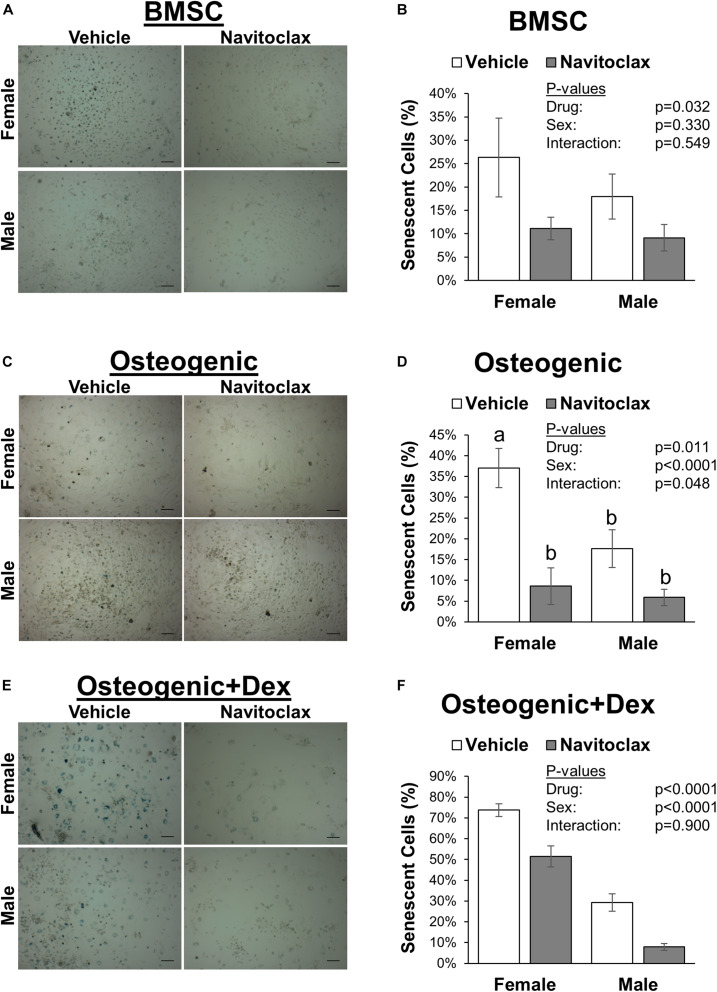
Navitoclax decreased senescent cell burden in BMSC and osteogenic cultures. Primary BMSC were harvested from 24 month old male and female mice and treated with navitoclax (5 μM) or vehicle (DMSO) for 5 days in BMSC growth medium **(A,B)**, osteogenic medium **(C,D)**, or osteogenic medium with 100 nM dexamethasone **(E,F)**. Senescent cells were identified by blue staining and quantified relative to total cell number. Representative images for each condition are shown in **(A,C,E)**. Scale bar: 100 μm. *P*-values shown represent statistical comparisons between groups, *p*-value for “drug” in 2-factor ANOVAs represents the comparison between navitoclax (gray) and vehicle (white)-treated cultures.

**FIGURE 3:**
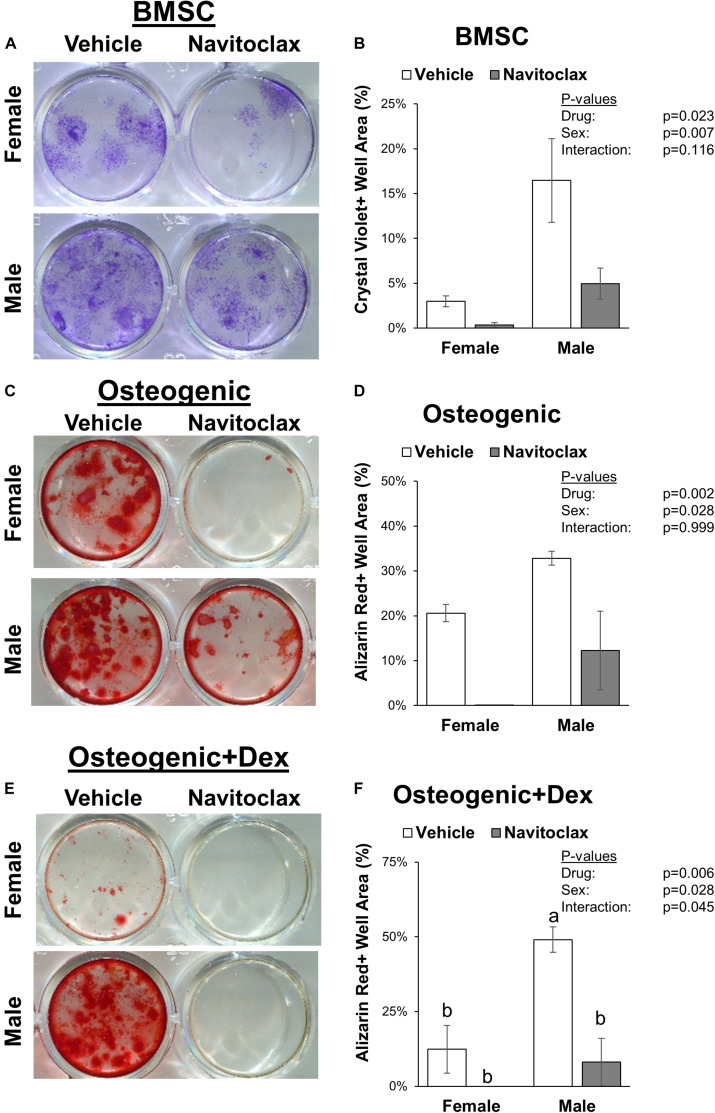
Navitoclax impaired colony formation and mineralized matrix production. Primary BMSC were treated with navitoclax (5 μM) or vehicle (DMSO) for 21 days in BMSC growth medium **(A,B)**, osteogenic medium **(C,D)**, or osteogenic medium with 100 nM dexamethasone **(E,F)**. Colony growth and matrix production were quantified as the fractional area of the well with staining after 21 days. Representative wells for each condition are shown in **(A,C,E)**. *P*-values shown represent statistical comparisons between groups, *p*-value for “drug” in 2-factor ANOVAs represents the comparison between navitoclax (gray) and vehicle (white)-treated cultures.

To test the direct effects of navitoclax on BMSC and osteoblastic function, we conducted mechanistic *in vitro* experiments. The senolytic effects of navitoclax were confirmed, as navitoclax generally reduced senescent cell burden, detected by the presence of SA-β gal staining, in BMSC and osteogenic cultures from both male (−49 to −73%) and female (−30 to −77%) cells (Figure 2). However, despite reducing senescent cell burden, BMSC from aged mice treated with navitoclax demonstrated a significant reduction (−89% female, −70% male) in colony formation as measured by crystal violet staining (Figures 3A,B). Similar to the effects seen in the primary cells from the mice treated *in vivo*, BMSC-derived osteoblasts treated with navitoclax *in vitro* were also impaired in their ability to produce a mineralized matrix under osteogenic culture conditions in the presence or absence of dexamethasone (Figures 3C–F), although pairwise comparisons did not reach statistical significance in females in the dexamethasone treated cultures, likely because mineralized matrix was substantially lower overall in the cultures from females as compared to males (Figure 3F).

**FIGURE 4:**
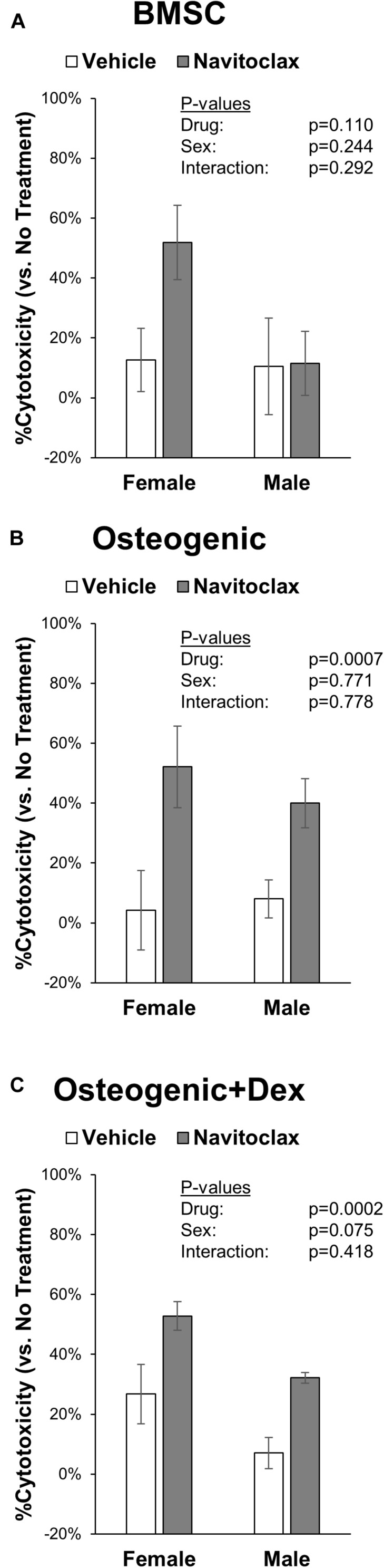
Navitoclax has cytotoxic effects in osteogenic cultures. Primary BMSC were treated with navitoclax (5 μM) or vehicle (DMSO) in BMSC growth medium **(A)**, osteogenic medium **(B)**, or osteogenic medium with 100 nM dexamethasone **(C)**. Cellular metabolic activity was quantified in MTT assays, and cytotoxicity for each treatment was calculated relative to untreated cells for each condition *P*-values shown represent statistical comparisons between groups, *p*-value for “drug” in 2-factor ANOVAs represents the comparison between navitoclax (gray) and vehicle (white)-treated cultures.

**FIGURE 5:**
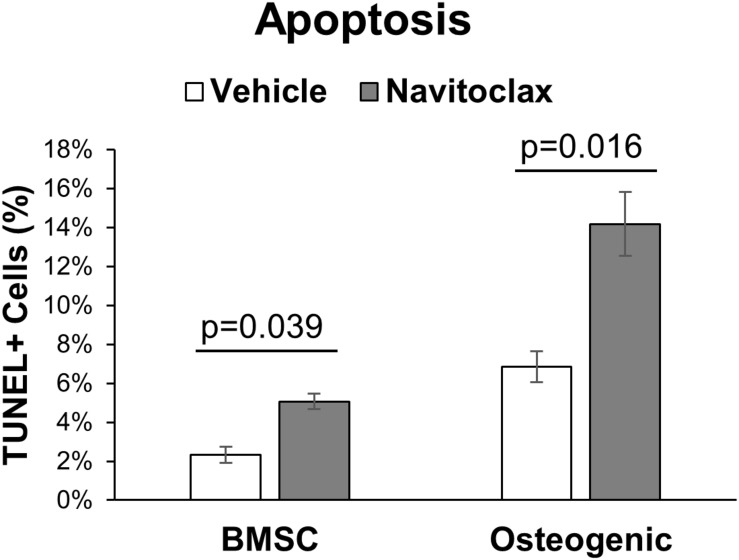
Navitoclax promotes apoptosis in BMSC and osteogenic cultures. Primary BMSC from aged female wildtype mice (27 months old) were treated with navitoclax (5 μM) or vehicle (DMSO) for 24 h in BMSC growth medium or osteogenic medium. Cells were stained with an *in situ* TUNEL detection kit to quantify apoptosis in the cultures; TUNEL+ cells were identified by green staining and normalized to total cell number. *P*-values shown represent pairwise comparisons between navitoclax (gray) and vehicle (white)-treated cultures.

With the goal of understanding why navitoclax impaired the growth and osteoblastic differentiation of BMSC and BMSC-derived osteoblasts, we conducted MTT assays to quantify relative cytotoxicity and TUNEL staining to quantify apoptosis. In MTT assays, navitoclax treatment did not significantly affect metrics of cytotoxicity in BMSC cultures (*p*_drug_ = 0.110, Figure 4A). In contrast, navitoclax significantly increased metrics of cytotoxicity in both male and female osteogenic cultures in the presence or absence of dexamethasone (+11.3 fold female osteogenic, +4.0 fold male osteogenic, +1.0 fold female osteogenic + dex, +3.6 fold male osteogenic + dex; Figures 4B,C). With regards to TUNEL staining, unfortunately, after BMSC and BMSC-derived osteoblasts were cultured on glass coverslips for 5 days in the presence of each treatment of interest, we detected no appreciable TUNEL staining in the cultures (data not shown). In a follow-up experiment, we harvested BMSC from aged female mice and subjected them to treatments of interest for the shorter timeframe of 24 h. These studies revealed that 24 h of navitoclax treatment significantly increased the fraction of TUNEL+ cells, indicative of apoptosis, in both BMSC (+117.8%) and osteogenic (+106.8%) cultures (Figure 5).

## Discussion

Senescent cells are not simply quiescent bystanders that accumulate during aging; rather, they instead have been described as “hyper-functional cells” (Blagosklonny, 2018) that can impair function of surrounding cell populations via release of secreted factors (SASP) and other mechanisms (Farr et al., 2016). Accordingly, senolytic therapies that target and remove these cells hold enormous promise for the treatment of age-related diseases, including osteoporosis (Baker et al., 2011; Chang et al., 2016; Farr et al., 2017; Grezella et al., 2018; Khosla et al., 2018; Geng et al., 2019; Kim and Kim, 2019). The senolytic agent navitoclax previously demonstrated efficacy in clearing senescent hematopoietic and skeletal muscle stem cells in mice, and importantly was shown to reduce senescence in both murine and human mesenchymal stromal cell populations (Chang et al., 2016; Zhu et al., 2016; Kim et al., 2017; Grezella et al., 2018). These observations suggested that navitoclax could hold promise as a treatment for age-related osteoporosis, but navitoclax’s effects on bone had not yet been reported *in vivo*. This led to the current study, with the goal of determining whether navitoclax would reduce senescent cell burden in murine stromal cell populations and accordingly improve bone mass in aged mice. Surprisingly, the opposite effect was observed *in vivo*; aged (24-month-old) male and female mice treated with navitoclax for 2 weeks developed trabecular bone loss in the proximal tibia attributable at least in part to impaired osteoprogenitor function.

To understand the mechanism behind this phenomenon, we examined osteogenic differentiation patterns of BMSC harvested from the treated mice, and also conducted *in vitro* treatment studies to determine navitoclax’s effects on metrics of apoptosis, cellular metabolic activity, and senescence in BMSC progenitors and in BMSC-derived osteoblasts. Osteogenic cultures were prepared with and without the addition of dexamethasone, a glucocorticoid that is commonly added to osteogenic cultures at low dosages (e.g., 100 nM) to enhance osteoblastic differentiation (Yamanouchi et al., 2001; Langenbach and Handschel, 2013; Yuasa et al., 2015), but can also affect osteoblastic cell metabolism and apoptosis particularly at higher concentrations (e.g., 1 μM) (Nie et al., 2018; Deng et al., 2019). In general, the effects of navitoclax were comparable between osteogenic cultures with and without dexamethasone (Figures 2–4). These *in vitro* studies revealed that while navitoclax was effective in targeting senescent BMSC and osteoblasts (Figure 2), it also drastically decreased BMSC colony formation and calcified matrix production by BMSC-derived osteoblasts (Figure 3), the latter of which was also observed in primary BMSC-derived osteoblast cultures from the mice that received navitoclax *in vivo*. These effects are somewhat surprising, because navitoclax is an inhibitor of the Bcl-2 family, and previous studies have shown that Bcl-2-deficient mice demonstrated enhanced osteoblastic differentiation and increased trabecular bone mass at young ages (Moriishi et al., 2014), whereas increased expression of Bcl-2 in mice induced apoptosis of osteocytes and reduced osteoblast differentiation (Moriishi et al., 2011). However, it is important to note that Bcl-2 knockout mice also demonstrated impaired osteoblast proliferation (evidenced by fewer bromodeoxyuridine-positive osteoblasts *in vivo* and decreased proliferation of primary osteoblasts *in vitro*) and increased osteoblastic apoptosis (Moriishi et al., 2014), suggesting the potential for deleterious effects on bone if the mice were able to survive for longer durations. This would be consistent with a previous human report that protein and mRNA levels of Bcl-2 were reduced in osteoblasts isolated from post-menopausal women with osteoporosis as compared to controls; this same cell population from osteoporotic patients also showed decreased proliferation and increased apoptosis, consistent with trends seen in Bcl-2-deficient mice (Pang et al., 2018). As navitoclax decreased BMSC colony formation and increased TUNEL staining of BMSC and BMSC-derived osteoblasts in the current study, these potential effects on Bcl-2-mediated mechanisms warrant further consideration in future studies.

While the exact molecular mechanism behind these deleterious effects on BMSC and osteoblasts is not yet known, it is notable that navitoclax demonstrated substantial cytotoxic effects in our studies, particularly in osteoblastic cultures (Figure 4). The dosage of navitoclax chosen for our studies was selected because this was the dosage reported to effectively deplete senescent murine stromal cells *in vitro* (Kim et al., 2017). Comparable doses did not appear to substantially impair survival of non-senescent IMR-90 myofibroblast cells, murine embryonic fibroblasts, or human preadipocytes (Zhu et al., 2016). Similarly, 5 μM navitoclax treatment for 72 h did not significantly affect MTT-based or trypan blue-based metrics of cell viability in non-senescent WI-38 human fibroblasts, although it did slightly but significantly decreased viability as measured by uptake of propidium iodide in the same cell line (Chang et al., 2016). Importantly, however, the *in vivo* dose selected for our studies was also chosen based on *in vivo* senolytic efficacy in a previous study with no reported side effects (Chang et al., 2016), whereas here we observed that this dose caused trabecular bone loss and impaired osteogenic differentiation of primary BMSC.

Many senolytic agents under investigation have shown a high degree of cell and tissue specificity. For example, navitoclax was previously reported to target senescent hematopoietic, mesenchymal, and muscle stem cells (Chang et al., 2016; Zhu et al., 2016; Grezella et al., 2018), but demonstrated minimal senolytic activity against primary human preadipocytes (Zhu et al., 2016). It is important to remember that senolytic agents like navitoclax are non-specific in nature; navitoclax, in particular, can act upon several different Bcl-2 family target proteins to promote apoptosis (Rudin et al., 2012; Souers et al., 2013; Zhu et al., 2016). It is possible that this promiscuity, or off-target effects, contributed to the negative effects we observed on bone *in vivo* and osteoblast cultures *in vitro*. Alternatively, there exists at least one report suggesting that the depletion of senescent cells *in vivo* could have detrimental consequences: promoting p16-driven senescence in beta cells of the pancreas improved glucose tolerance in diabetic mice, and both artificial and natural age-related increases in p16-driven senescence in pancreatic beta cells increased glucose-stimulated insulin production, suggesting that depletion of these cells (via senolytic targeting) could promote a diabetic phenotype (Helman et al., 2016; Blagosklonny, 2018). In either case, it is important to stress that the results obtained in the current study are specific to navitoclax, and should not be broadly inferred to relate to other senolytic therapeutics. In addition, we acknowledge that our endpoints in the current study were limited; for example, we did not conduct an in-depth investigation of bone mass across several skeletal sites (e.g., lumbar vertebrae, cortical bone density) in these mice, nor use more powerful techniques like micro-computed tomography to assess bone mass with high resolution. Therefore, these results should be considered preliminary, and should be confirmed by a more in-depth and extended study of the effects of navitoclax on the skeleton *in vivo.* However, the consistency observed between navitoclax’s *in vivo* effects (trabecular bone loss and impaired BMSC-derived osteoblast differentiation and function) and *in vitro* effects (impaired BMSC colony formation and BMSC-derived osteoblast matrix production, impaired cellular metabolic activity in MTT assays for BMSC and BMSC-derived osteoblasts) do suggest a consistent and potentially harmful effect on skeletal-lineage cells that should be noted and explored further.

In conclusion, while navitoclax has demonstrated promise as a senolytic agent for the skeletal system via *in vitro* studies, the current work suggests that navitoclax’s *in vivo* efficacy for treating age-related bone loss may be limited. It is important to emphasize that these small-scale murine studies reported here are only an initial glimpse into navitoclax’s *in vivo* effects; larger-scale studies, in rodents and larger animal models, are needed to definitively assess navitoclax’s potential as a therapeutic agent to combat age-related musculoskeletal dysfunction and bone loss.

## Data Availability Statement

The datasets generated for this study are available on request to the corresponding author.

## Ethics Statement

The animal study was reviewed and approved by the Institutional Animal Care and Use Committee at Augusta University.

## Author Contributions

All authors meet authorship criteria, and have read and approved the final submitted manuscript. MH and MM-L: study design. AS, RR, RB, JP, KY, and MM-L: data collection. AS and MM-L: initial manuscript preparation. AS, MH, and MM-L: final manuscript preparation.

## Conflict of Interest

The authors declare that the research was conducted in the absence of any commercial or financial relationships that could be construed as a potential conflict of interest.
